# Simple surgical explant technique for the EDWARDS INTUITY rapid deployment valve: a case report of prosthetic valve endocarditis

**DOI:** 10.1186/s44215-025-00203-8

**Published:** 2025-03-28

**Authors:** Hironobu Sakurai, Naonori Kawamoto, Satoshi Kainuma, Kota Suzuki, Takashi Kakuta, Masaya Hirayama, Satsuki Fukushima

**Affiliations:** Department of Cardiac Surgery, National Cerebral and Cardiovascular Center, Suita, Osaka Japan

**Keywords:** Rapid deployment valve, EDWARDS INTUITY valve, Valve replacement, Infective endocarditis

## Abstract

**Background:**

A rapid deployment valve can shorten operation times and improve hemodynamics. However, explantation can be challenging because of the unique structure of such valves, including an inflow frame covered by textured sealing cloth beneath the sewing cuff. In this case, we report a simple explantation technique.

**Case presentation:**

This case involved a 79-year-old woman with prosthetic valve endocarditis who had undergone aortic valve replacement with a rapid deployment valve 5 years earlier. Preoperative echocardiography revealed severe mitral regurgitation and a highly mobile mass on the posterior leaflet. The prosthetic valve had thickened cusps without regurgitation. Emergent surgery was performed to explant the prosthetic valve and replace both the aortic and mitral valves through a re-median sternotomy under routine cardiopulmonary bypass support. The textured sealing cuff was detached from the surrounding tissue after separating the rigid outflow portion from the transformable inflow portion by cutting the fabric. No annular or sub-annular damage was observed. *Enterococcus faecalis* was cultured from the blood. The patient received 6 weeks of antimicrobial therapy and was discharged without symptoms of heart failure or infection.

**Conclusion:**

The patient successfully underwent valve explantation and double valve replacement for prosthetic valve endocarditis. This method is safe and feasible for explanting rapid deployment valves with minimal tissue damage.

## Background

Rapid deployment valves are widely implanted because of their ability to shorten operation times and improve hemodynamics [1–4]. Although these valves have been reported to achieve good outcomes, some cases require explantation because of prosthetic valve dysfunction or infection [1, 2]. The valve has a unique structure, with an inflow frame covered by textured sealing cloth under the sewing cuff to minimize paravalvular leakage, which can make explantation more challenging. However, only a few articles have described explantation techniques for rapid deployment valves [5]. We herein report a simple explantation technique for a rapid deployment valve 5 years after implantation in a patient with prosthetic valve endocarditis.

## Case presentation

### Patient

**Fig. 1:**
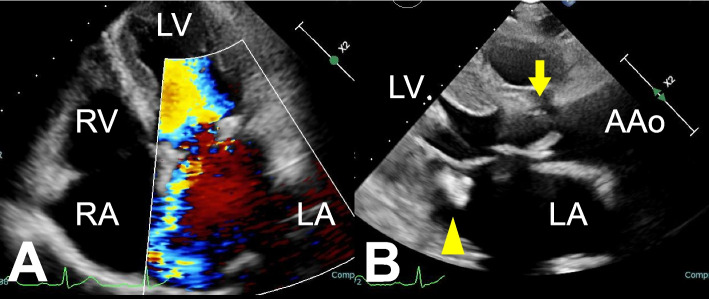
Preoperative transthoracic echocardiography. **A** Severe mitral regurgitation due to the posterior leaflet prolapse. **B** A 12-mm highly mobile mass was present on the posterior leaflet (yellow arrowhead), and the INTUITY aortic valve had thickened cusps without regurgitation (yellow arrow). AAo, ascending aorta; RA, right atrium; LA, left atrium; RV, right ventricle; LV, left ventricle

A 79-year-old woman had a history of hypertension and chronic glomerulonephritis, which had required hemodialysis for 14 years. She underwent aortic valve replacement (AVR) using a 19-mm EDWARDS INTUITY valve (Edwards Lifesciences, Irvine, CA, USA) at the age of 74 years. After 2 months of antimicrobial therapy for prolonged fever, she was diagnosed with infective endocarditis and referred to our hospital. *Enterococcus faecalis* was cultured from her blood. Preoperative echocardiography revealed severe mitral regurgitation due to posterior leaflet prolapse (Fig. 1A) and a 12-mm highly mobile mass on the posterior leaflet (Fig. 1B). The INTUITY aortic valve had thickened cusps without regurgitation (Fig. 1B). Preoperative computed tomography showed bilateral pleural effusion and ascites, with no evidence of embolism. Emergent surgery, as described below, was performed, followed by 6 weeks of antimicrobial therapy with ampicillin and gentamicin. Postoperative echocardiography showed no vegetation and well-functioning prosthetic double valves with a good ejection fraction. The patient was discharged without symptoms of heart failure or infection.

### Surgical procedure

**Fig. 2:**
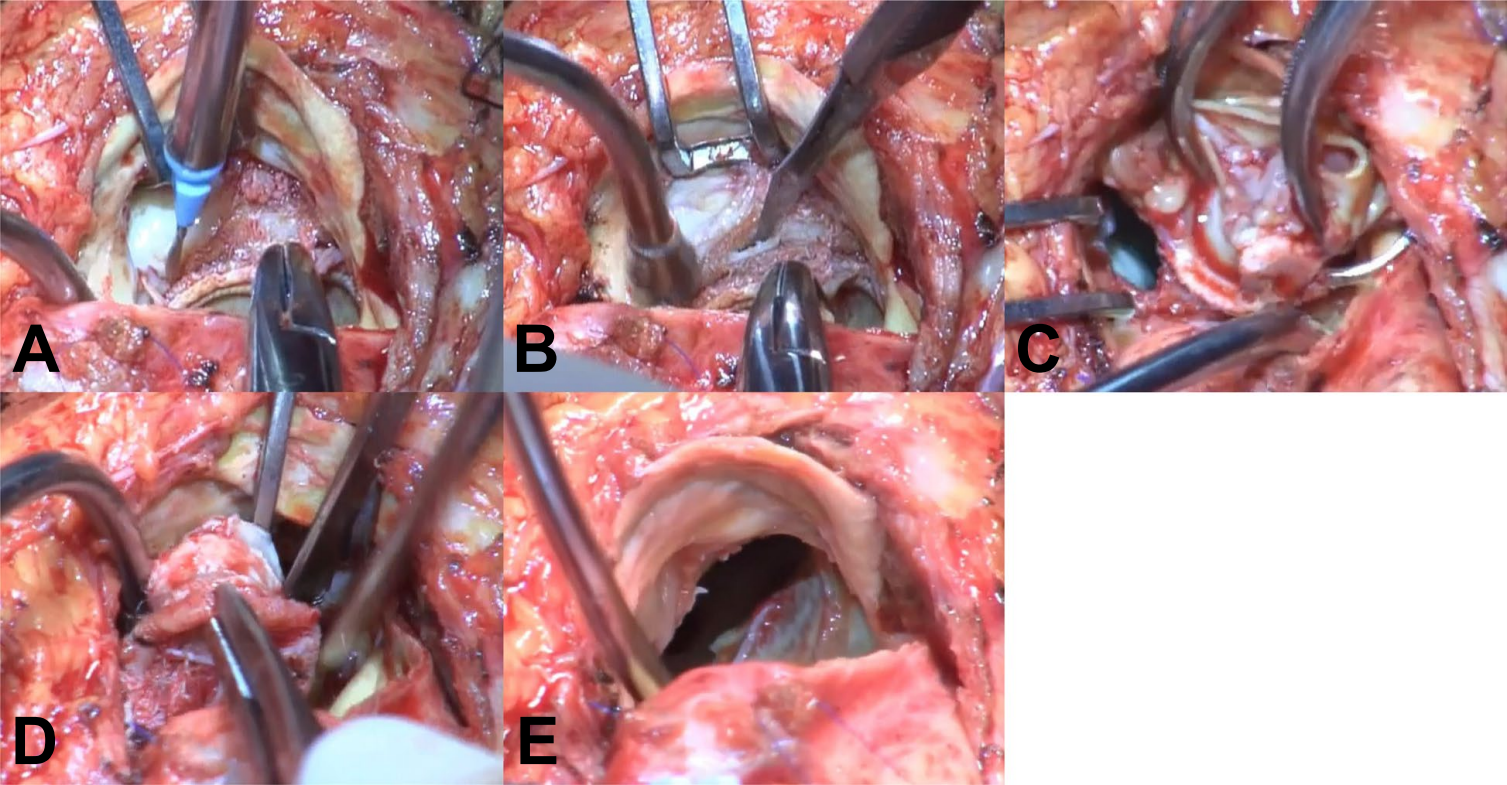
Intraoperative photographs. **A** The pannus that was completely covering the sewing cuff was cleared from the sewing ring using a beaver blade. **B** A No. 11 blade was easily inserted between the rigid outflow portion and the inflow portion. The INTUITY valve was explanted by separating the rigid outflow portion from the transformable inflow portion by **C** cutting the fabric and **D** detaching the textured sealing cuff from the native surrounding tissue. **E** There was no annular or sub-annular damage

**Fig. 3:**
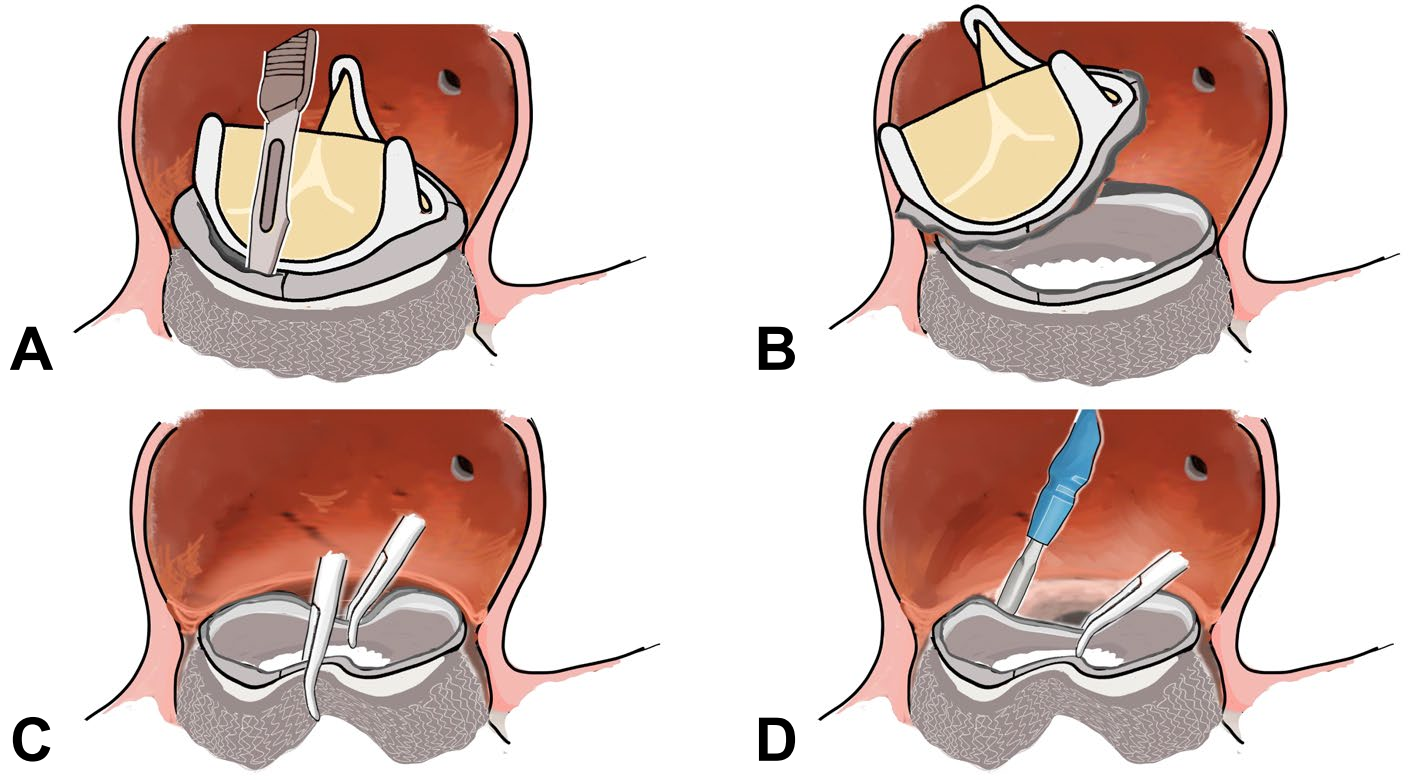
Schema of the surgical technique to explant the INTUITY valve. **A**, **B** The outflow portion was easily separated from the inflow frame using a No. 11 scalpel. **C** The inflow frame was pulled away by pushing inward. **D** The frame was then carefully detached from the native surrounding tissues using a No. 11 blade or beaver blade

Emergent surgery was performed through a re-median sternotomy under routine cardiopulmonary bypass support. A vent was placed via the right upper pulmonary vein. After aortic cross-clamping and the application of antegrade cardioplegia, vegetation was found around the aortic prosthesis as well as the mitral valve via left atriotomy. A transverse aortotomy was also performed to expose the infected INTUITY rapid deployment valve. Vegetation was found on the leaflet of degenerated INTUITY valve without any infiltration into annulus. The INTUITY valve was completely covered by pannus, creating severe adhesion between the device and the contacting native tissue. The pannus was cleared from the sewing ring using a beaver blade (Fig. 2A). Because a metal wire was absent and only fabric was present between the outflow portion and inflow frame, we could easily separate the outflow portion from the inflow frame using a No. 11 scalpel (Figs. 2B, C and 3A, B).

Similar to the double Kocher clamp technique [6], two forceps (or Kocher clamps) were applied to push the inflow frame inward, pulling it away from the native tissue en-bloc (Fig. 3C). Adhesions between the inflow frame and the surrounding native tissue were dissected using a No. 11 blade or a beaver blade (Figs. 2D and 3D). After complete removal of the rapid deployment valve, the infected mitral valve leaflet was resected, and the infected native tissue was debrided. Since no annular or sub-annular damage was observed after removal of INTUITY valve, annular reconstruction was not necessary (Fig. 2E). Conventional AVR and mitral valve replacement were performed using a 19-mm Avalus Ultra valve (Medtronic, Minneapolis, MN, USA) and a 27-mm Epic mitral valve (St. Jude Medical, St. Paul, MN, USA). The operation time and cardiac arrest time were 384 min and 125 min, respectively.

## Discussion and conclusions

Recently, rapid deployment valves, which shorten operation times and demonstrate good hemodynamic behavior resulting in favorable long-term outcomes [1–4], have been widely implanted during AVR. With the increasing number of AVRs using rapid deployment valves, the demand for explantation is expected to rise in the future [1, 2]. Coti et al. [1] reported a 91% rate of 7-year freedom from re-intervention or re-operation after rapid deployment valve implantation. Transcatheter AVR (TAVR) in surgical AVR (SAVR) procedures is an effective alternative for patients with prosthetic valve failure. However, TAVR in SAVR may not be feasible for some patients because of infection or unfavorable anatomy. Furthermore, TAVR in SAVR for rapid deployment valves is not yet covered by insurance in Japan. As a result, explantation of the rapid deployment valve remains the only feasible option in such cases.

The valve has a unique structure. The outflow portion, including the stent strut and leaflet, was rigid. By contrast, the balloon-expandable inflow frame consisted of a transformable stainless frame covered by textured sealing cloth. Because of its structural characteristics, significant adhesion between the textured sealing cloth under the sewing cuff and the surrounding tissue is expected, which complicates valve explantation. The adhesion would be particularly severe in cases of no annular infection.

Uehara et al. [5] published the first report of a surgical explantation technique for the INTUITY valve, addressing moderate paravalvular leakage and hemolysis by employing an elevatorium and a hook. In that case, the INTUITY valve was explanted 1 year after implantation. Simple blunt dissection between the native aortic annulus and the sewing cuff, achieved by shaving with the elevatorium and carefully bending the inflow frame inward using a hook, successfully separated the prosthesis from the surrounding tissue.

In our case, because 5 years had passed since valve implantation, the sewing cuff was completely covered with pannus, and there were no gaps between the prosthesis and the native annulus. Considering the severe adhesion of the inflow portion covered by textured sealing cloth, we separated the rigid outflow portion from the transformable inflow position to create enough space and pushed the inflow frame toward the center and away from the native tissue similar to the double Kocher clamp technique [6]. We believe this method is safe and feasible in any case to remove the rapid deployment valve with minimal damage to the surrounding tissues, including the ventricular septum, the native valve annulus, and the mitral valve leaflet.

## Data Availability

The datasets used and/or analyzed during the current study are available from the corresponding author on reasonable request.

## Abbreviations

AVR: Aortic valve replacement
TAVR: Transcatheter aortic valve replacement
SAVR: Surgical aortic valve replacement
